# Enhanced Electroluminescence via a Nanohybrid Material Consisting of Aromatic Ligand-Modified InP Quantum Dots and an Electron-Blocking Polymer as the Single Active Layer in Quantum Dot–LEDs

**DOI:** 10.3390/nano12030408

**Published:** 2022-01-26

**Authors:** Hyung-Seok Choi, Silvia Janietz, Vladimir Roddatis, Andre Geßner, Armin Wedel, Jiyong Kim, Yohan Kim

**Affiliations:** 1 Functional Polymer Systems, Fraunhofer Institute for Applied Polymer Research (IAP), Geiselbergstrasse 69, 14476 Potsdam, Germany; hyung.seok.choi@iap.fraunhofer.de (H.-S.C.); silvia.janietz@iap.fraunhofer.de (S.J.); andre.gessner@iap.fraunhofer.de (A.G.); armin.wedel@iap.fraunhofer.de (A.W.); 2 GFZ German Research Center for Geosciences, Helmholtz Centre Potsdam, Telegrafenberg, 14473 Potsdam, Germany; vladimir.roddatis@gfz-potsdam.de

**Keywords:** InP quantum dot, aromatic surface ligands, electron blocking material, π–π interaction, nanohybrid

## Abstract

Electron overcharge causes rapid luminescence quenching in the quantum dot (QD) emission layer in QD light–emitting diodes (QD–LEDs), resulting in low device performance. In this paper we describe the application of different aromatic thiol ligands and their influence on device performance as well as their behavior in combination with an electron blocking material (EBM). The three different ligands, 1–octanethiol (OcSH), thiophenol (TP), and phenylbutan–1–thiol (PBSH), were introduced on to InP/ZnSe/ZnS QDs referred to as QD–OcSH, QD–TP, and QD–PBSH. PBSH is in particular applied as a ligand to improve QD solubility and to enhance the charge transport properties synergistically with EBM probably via π–π interaction. We synthesized poly-[N,N-bis[4-(carbazolyl)phenyl]-4-vinylaniline] (PBCTA) and utilized it as an EBM to alleviate excess electrons in the active layer in QD–LEDs. The comparison of the three QD systems in an inverted device structure without the application of PBCTA as an EBM shows the highest efficiency for QD–PBSH. Moreover, when PBCTA is introduced as an EBM in the active layer in combination with QD–PBSH in a conventional device structure, the current efficiency shows a twofold increase compared to the reference device without EBM. These results strongly confirm the role of PBCTA as an EBM that effectively alleviates excess electrons in the active layer, leading to higher device efficiency.

## 1. Introduction

The Restrictions of Hazardous Substances (RoHS) directive limits the concentration of hazardous materials in electrical equipment and thus also affects the usage of Cd–based QDs in display technologies. InP QDs have been identified as a potent replacement candidate due to their intriguing optical properties, such as outstanding photoluminescence quantum yield (PL QY), tunable emission spectrum and high color purity [1,2]. In particular, the cutting-edge PL QY of over 90% from InP QDs as green and red emitters has led to great success of the QD enhancement film (QDEF) technology employed on top of the blue-backlight unit in the display industry [3]. Furthermore, QD–LEDs have been continuously studied as the next-generation display technology which is expected to show higher color purity, deeper contrast, and higher energy efficiency compared to the conventional QDEF liquid crystal display [4]. Recently, Won, Y et al. reported high performance electroluminescent (EL) devices having an incredibly high external quantum efficiency (EQE) of more than 20% by using state-of-the-art InP QDs. In their research, hexanoic acid-capped QDs were inserted as active layer between an organic hole transport layer (HTL) and an inorganic electron transport layer (ETL) [5].

In order to achieve a commercially feasible QD–LED, many studies were performed to understand the mechanisms regarding the interaction between QDs and charge transport layers [6,7,8]. During the operation of QD–LEDs, the QD active layer is generally affected by a complex combination of electric field effects and carrier charging across the device, leading to different quenching mechanisms, such as the field induced quenching, [9,10], interfacial charge transport [11], and charged QDs by excess charge injection [12]. In particular, the imbalance of charge injection and transport in the active layer can give rise to the accumulation of charges in the QD layer. These generated excess charges in the active layer can lead to the aforementioned non-radiative processes resulting in a decreased efficiency of QD–LEDs [10]. These issues can be addressed by different strategies. On the one hand, core/shell based QD materials can be designed with an elaborately modified outer shell, such as a gradient or giant shell including the surface ligands. The optimization of the QD structure can reduce non-radiative Auger processes or introduce strong confinement effects to mitigate field-induced quenching [13,14]. Furthermore, thiophenol (TP) ligands were previously reported to provide better charge carrier transport properties for Cd-based QD–LED applications [15]. On the other hand, the balancing of charge carriers in the active layer through the design of device structure provides powerful yet complex opportunities to elevate the device performance. One of the most promising methods to elevate the device performance is the introduction of an electron-blocking layer (EBL) to balance charge carriers in the active layer which has already been demonstrated for Cd-based QD–LEDs. For instance, Dai et al. reported that the accumulation of electrons in the QD active layer that is caused by the imbalanced charge carrier mobility can be addressed by the implementation of an EBL. In their device configuration, the electron mobility of the ZnO nanoparticle (NP) ETL (1.8 × 10^−3^ cm^2^V^−1^s^−1^) was much higher than the hole mobility of the poly-(N,N’-bis-4-butylphenyl-N,N’-bisphenyl)benzidine (poly-TPD, 1 × 10^−4^ cm^2^V^−1^s^−1^) HTL. They showed that a 6 nm thick poly(methyl methacrylate) (PMMA) interlayer can be utilized as an EBL between the active layer and the ZnO NP ETL in a Cd-based QD–LED structure to improve the lifetime to over 100,000 h at 100 cd/m^2^ with a very high EQE of 20.5% [16]. M. Rahmati et al. described a novel device design for conventional structured Cd-based QD–LEDs that incorporates multiple PMMA EBLs inserted in between multiple QD layers, which reduces electron leakage from the active QD layer. This design results in significantly increased radiative recombination in the active QD layers, showing the extremely high performance with a current efficiency of 17.8 cd/A and luminance of 194,038 cd/m^2^ [17]. Yan fu et al. developed an inverted Cd-based QD–LED structure incorporating an active QD layer sandwiched by ultrathin double charge-blocking–layers using a poly(9–vinlycarbazole) (PVK) layer to prevent accumulation of excess electrons, and a polyethylenimine ethoxylated (PEIE) layer to reduce the hole-injection barrier. This structure improved the charge imbalance in the active layer and showed highly enhanced performance with a current efficiency of 89.8 cd/A and a maximum brightness of 72,814 cd/m^2^ [18]. However, although conventional EBLs can induce charge carrier balance through the suppression of electron injection, they need to be processed in a way that forms a layer with a reproducible and homogeneous thickness. Non-uniform EBL thickness in the QD–LED may have a detrimental impact on device efficiency. In addition, a homogeneous morphology is relatively difficult to obtain via the widely used spin–coating process. Furthermore, the introduction of one or multiple extra layers also requires additional processing steps, increasing process complexity and thus reducing reproducibility. In addition, PMMA or similar materials are not the most suitable solution due to their purely insulating properties. To overcome these challenges, a hybrid active layer composed of QDs dispersed in a semiconducting polymer matrix can be applied. To achieve a good dispersion of the QDs in the QD/EBM polymer hybrid material the application of a chemically modified polymer may be realized, where its functional groups bind to the QD surface. Bae et al. showed that a well–dispersed QD/HTL hybrid active layer composed of a grafted polymer on the QD surface contributes to the suppression of efficiency roll-off at high current density, resulting in an enhanced charge carrier balance in the QD–LEDs [19]. Fokina, A et al. utilized QDs blended with a side-chain polymer with disulfide groups as the active layer to achieve enhanced device performance with EQE of 6.09% [20].

In this research, we propose a QD/EBM polymer hybrid active layer formed by poly-[N,N-Bis [4-(carbazolyl)phenyl]-4-vinylaniline] (PBCTA) [16] blended with surface-modified InP QDs. This is possible because the lowest unoccupied molecular orbital (LUMO) of PBCTA was found to be high enough (−1.9 eV) to obstruct electron injection from the ETL to the active layer. We aimed at introducing π–π interactions via the aromatic substructure which are widely known to provide a good compatibility [21] and charge transport channel between the QD surface and an EBL with a semiconducting polymer structure and properties [22]. For that purpose, we investigated thiophenol (TP) and 4-phenylbutane-1-thiol (PBSH) as ligands on the QD surface (QD–PBSH) to facilitate smooth charge transport between the QDs and the EBM PBCTA at the interfacial regime in the hybrid active layer. These QD systems were compared to the QDs with aliphatic octanethiol (OcSH) ligands. Two well-known types of device structure, inverted and conventional, were applied in our investigations. The inverted structure was used to investigate the three different QDs, QD–OcSH, QD–PBSH and QD–TP, because it provides a good orthogonal solvent process that avoids interface mixing between QDs and the underlying ZnO NP layer [23]. To investigate the influence of the QD/EBM nanohybrid material on device performance, a particularly designed conventional device structure utilizing 2,2′,2″-(1,3,5-benzinetriyl)-tris(1-phenyl-1-H-benzimidazole) (TPBi) as an ETL was applied to intentionally create an electron overcharge in the active QD layer. TPBi is known to favor an accumulation of electrons in the QD layer even though it provides significantly lower electron mobility (3.8–8.0 × 10^−5^ cm^2^V^−1^s^−1^) [6] compared to ZnO NPs. Two effects are responsible for this behavior. First, the electron injection is favorable considering the energy band alignment between the LUMO of TPBi and the conduction band minimum (CBM) of InP-based QDs. Second, ZnO NP not only provides high electron mobility but also enables additional electron extraction pathways. Due to the similarity of the energy level of mid-band gap states of ZnO NPs and the trapped electrons in the QD layer, electrons can be extracted from the QD layer back into the ZnO NP layer [24]. This process alleviates the electron overcharging induced by the high electron mobility of ZnO NP at least partially. 

The application of QD–PBSH and polymer PBCTA nanohybrid material as the active layer in a QD–LED leads to improved device efficiency, confirming a synergistic effect between conjugated QD ligands and EBM PBCTA. High-angle annular dark–field scanning transmission electron microscope (HAADF–STEM) imaging was performed on the cross-section of the QD/EBM polymer hybrid layer in the QD–LED structure to investigate morphology. The results showed evidence for a reduced phase separation in the hybrid active layer with QD–PBSH as compared to that with QD–OcSH.

## 2. Materials and Methods

### 2.1. Reagents

Since many abbreviations of chemicals were used in manuscript, the basic information of the chemicals is listed at the Table 1.

### 2.2. Synthesis of 4-Phenylbutan-1-Thiol (PBSH)

#### 2.2.1. Synthesis of S-(4-Phenylbutyl) Ethanethiate (PBET)

Firstly, 5 mL (33.3 mmol) of PhBe and 3.6 mL (50 mmol) of TA were weighed into a reaction quartz flask with a septum. The apparatus was evacuated and backfilled with argon three times, and 50 mL of anhydrous CHCl_3_ were injected via the septum. The mixture was stirred and irradiated with a high-pressure mercury lamp for 1 h. The reaction mixture was cooled to room temperature, and afterward washed with water. The water phase was again extracted with CHCl_3_. The collected organic phases were dried with Mg_2_SO_4_, filtered, and concentrated using a rotatory evaporator. Flash column chromatography was carried out with heptane with a gradient to n–HEP/EA 3:1, 80 mL/min led to 5.82 g (84.8%) of the pure product. ^1^H NMR (500 MHz, CDCl_3_, d, ppm) 7.33 –7.26 (m, 4H), 7.24 –7.16 (m, 3H), 2.92 (t, J = 7.2, 2H), 2.64 (t, J = 7.5, 2H), 2.34 (s, 3H), 1.75–1.59 (m, 5H).

#### 2.2.2. Synthesis of PBSH

Previously synthesized 2 g (9.7 mmol) of S-(4-phenylbutyl)ethanthioate (PBET) was solved in 20 mL toluene in a reaction flask with a septum. The apparatus was evacuated and backfilled with argon three times. Next, 13.5 mL (164.8 mmol) of propylamine and 149.4 mg (0.97 mmol) of dithiothreitol were introduced into the reaction flask. The reaction mixture was stirred and heated up to 50 °C for one hour, cooled down to room temperature, extracted with water three times, dried with MgSO_4_, and the toluene was evaporated. The crude product was purified by flash column chromatography with heptane/ethyl acetate 3:1 and 1.58 g (98%) of the clean product was isolated. ^1^H NMR (500 MHz, CDCl_3_, d, ppm): 7.32–7.24 (m, 2H), 7.23–7.16 (m, 3H), 2.63 (t, J = 7.4, 2H), 2.60–2.51(m, 2H), 1.79–1.61 (m, 4H), 1.34 (td, J = 7.7, 1.5, 1H)

### 2.3. Synthesis of Poly-[N,N-Bis [4-(carbazolyl)phenyl]-4-vinylaniline] (PBCTA)

**Figure 1:**
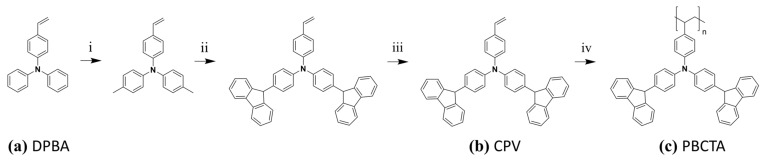
Synthetic scheme for (**a**)DPBA, (**b**) CPV, (**c**) PBCTA, and reagents and conditions: (i) CHCOOH, H_2_O 10:1, KI, KIO_3_, 3 h, 80 °C (ii) K_2_CO_3_, activated Cu–Bronze, 18–crown–6, 1,2–dichlorobenzene, 48 h, 180 °C (iii) KOt–Bu, MePPh_3_Br, THF, 2 h, 0 °C (iv) THF, AIBN, 50 °C, 60 h; CHCOOH, H_2_O 10:1, KI, KIO_3_, 3 h, 80 °C (ii) K_2_CO_3_, activated Cu–Bronze, 18–crown–6, 1,2–dichlorobenzene, 48 h, 180 °C (iii) KOt–Bu, MePPh_3_Br, THF, 2 h, 0 °C (iv) THF, AIBN, 50 °C, 60 h.

The monomer N,N-Bis[4-(carbazolyl)phenyl]-4-vinylaniline (CPV, Figure 1b) was synthesized in a four–step synthesis starting from 4-(diphenylamino)-benzaldehyde (DPBA, Figure 1a) according to the description in previous report [25]. The polymerization was carried out in a glove box system under nitrogen atmosphere, where THF acts as solvent while the initiator was AIBN. The polymerization temperature was kept at 50 °C for 60 h. The corresponding PBCTA (Figure 1c) was isolated in a yield of 60% with M_w_ of 14.000 g/mol and M_n_ of 3.400 g/mol, polydispersity index (PDI) value 4.1, and a glass transition temperature of 247 °C. ^1^H NMR (500 MHz; CDCl_3_) [ppm] δ 8.40–7.60 (aromatic) 7.60–6.78 (aromatic); 2.60–0.40 (backbone); elemental analysis: C 86.52; H 5.68; N 6.33; calculated: C 87.82; H 5.19; N 6.98).

### 2.4. Synthesis of Zinc Octanoate (Zn(OctOAc)_2_)

In a 500 mL round bottom flask, 0.12 mol (9.768 g) of ZnO and 0.32 mol (76.3 mL) of 1–OctOAc were added and suspended throughout the 300 mL of EtOH with vigorous stirring and kept for a couple of hours until the suspension became a clear solution. After being cooled down to room temperature, the obtained Zn(OtcOAc)_2_ was filtered and washed with pure ethanol twice to remove unreacted 1–OctOAc.

### 2.5. Synthesis of InP/ZnSe/ZnS Quantum Dots with Different Thiol Ligands

**Figure 2:**
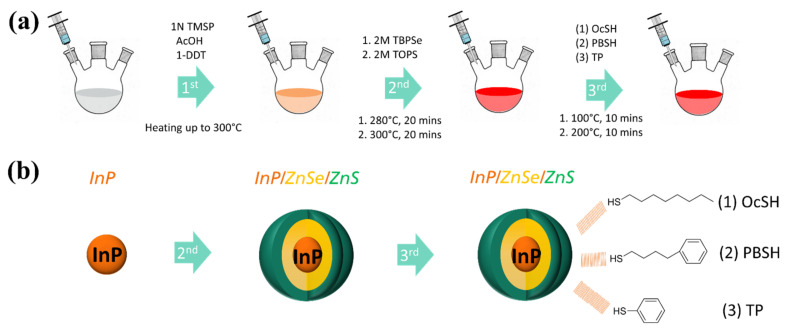
(**a**) The reaction scheme of InP/ZnSe/ZnS QDs and (**b**) the expected structure of QD at each step. QD–OcSH, QD–PBSH, and QD–TP from in situ ligand modification with (1) OcSH, (2) PBSH, and (3) TP ligands.

The synthesis procedure followed a previously reported method with a slight modification [26]. The reaction scheme and ligand exchange process are described in Figure 2. 1 mmol (292 mg) of In(OAc)_3_, 2 mmol (706 mg) of Zn(OctOAc)_2_ and 10 mL of 1–ODE was weighed into a 50 mL 3-neck flask. All precursors were heated up to 160 °C under an argon atmosphere until a clear yellowish-brown melt was formed. The mixture was then cooled down to 100 °C and 60 µL of AcOH, 120 µL of 1–DDT and 1 mL of P(TMSi)_3_ (1M in 1–ODE) were rapidly injected with strong stirring, heated up to 300 °C and kept for 20 min for further growth of the InP core. The as-prepared core solution was cooled down to 100 °C and 4 mmol (734 mg) of Zn(OAc)_2_ was added into a flask with argon reflux and stirred for 9 min. Next, TBP–Se (2M) was injected rapidly, and the reaction temperature was increased to 280 °C and maintained for 20 min for the additional ZnSe shelling. Next, 1 mmol of TOP–S (2M) was injected at 300 °C and reacted for 20 min for the ZnS shell. After the reaction was completed, the crude solution was cooled down to 100 °C to be prepared for the following ligand modification process. 0.5 mmol of OcSH, PBSH and TP were prepared for QD–OcSH, QD–PBSH QD–TP, respectively. Each thiol ligand was introduced into separate flasks with 5 mL crude solution, and the mixture was stirred vigorously for 10 min at 100 °C, followed by increased reaction temperature to 200 °C kept for 10 min. After the reaction, the QD solution was purified with AC 3 times with centrifugation at 4000 rpm.

### 2.6. Inverted Structure of Quantum Dot Light-Emitting Diode (QD–LED) Fabrication

First, the indium tin oxide (ITO) substrates were cleaned by sequential ultra–sonication with AC, MeOH, and IPA and dried in an oven, followed by the oxygen plasma treatment. The following steps were performed in a glove box under nitrogen atmosphere. The ZnO NP ETL prepared using procedures previously reported in the literature [27] were spin–coated onto the cleaned ITO substrates at 3000 rpm for 30 s as an ETL, followed by drying at 180 °C for 30 min. Then, the QDs, dispersed in toluene with a concentration of 3 mg/mL, were spin–coated onto the ZnO NP layer at 3000 rpm for 30 s without further thermal annealing as an active emission layer. After the solution processes, TCTA (40 nm) and MoO_3_/Ag (5 nm/150 nm) were deposited by the thermal evaporation on the QD layer in a high-vacuum chamber as a HTL and metallic anode, respectively.

### 2.7. Conventional Structure QD–LED Fabrication

First, the ITO substrates were cleaned by the same possess as described above in the inverted structure fabrication. PEDOT:PSS as a hole injection layer was spin-coated on the ITO substrates at a spin rate of 3000 rpm for 30 s in normal air atmosphere, and transferred into a glove box under nitrogen atmosphere for annealing on a hot plate at 180 °C for 30 min. Then, the poly–TPD (3 mg/mL in in chlorobenzene) as a HTL was also spin-coated at a spin rate of 4000 rpm for 1 min on top of the PEDOT:PSS layer and dried on the hot plate at 130 °C for 30 s. In order to deposit the active emission layer, the colloidal QDs (5 mg/mL in toluene, ca. 10 nm) or the QD/EBM hybrid solution 1:1 weight ratio, 10 mg/mL in toluene, approximately 30 nm) were spin-coated onto the poly–TPD layer at a spin rate of 3000 rpm for 20 s. After the solution processes conducted in an N_2_ atmosphere, TPBi (65 nm) and Ca/Ag (30 nm/150 nm) were deposited on the QD layer by thermal evaporation as an ETL and metallic cathode, respectively.

### 2.8. Characterization

The photoluminescence (PL) spectrum and absolute photoluminescence (PL) quantum yield (QY) of QDs were recorded using an integrating sphere with a Hamamatsu C9920–02 system (Hamamatsu, Japan) including 150 W Xenon lamp, monochromator, and back–thinned CCD multichannel analyzer. The UV–Vis spectrum was obtained over a wavelength range of 250 to 800 nm using a PerkinElmer Lambda 19 spectrometer (Waltham, MA, USA). For these measurements, the optical density of the QD samples in the respective solvent (usually toluene) was set to 0.1. The excitation wavelength was set to 350 nm. Time-correlated single photon counting measurements were performed on a FLS920-stm fluorescence spectrometer (Edinburgh Instruments, EI, Livingston, UK). An EPLED-320 diode laser (Edinburgh Instruments, EI, Livingston, UK) with an emission wavelength of 405.6 nm (bandwidth 12.2 nm), a pulse width of 628.3 ps and a pulse period of 500 ns used as excitation source. Measurement points were collected on a 200 ns scale distributed over 512 channels until peak intensity reached 10,000 counts. Fourier transform infrared spectroscopy (FT–IR) was measured by a Thermo Scientific Nicolet iS20 FTIR Spectrometer (Waltham, MA, USA) with an attenuated total reflection (ATR) hemisphere. The ionization energy was measured by a photoelectron spectrophotometer (AC–2, Riken Keiki) (Tokyo, Japan) under air in the incident energy range of 3.4 eV to 6.2 eV. Current density–voltage–luminance (J–V–L) characteristics were measured with an experimental setup consisting of a source meter (Keithley SMU 236, voltage source range: ±11.000 V, step size: 1 mV, accuracy: ± (0.033% + 2.4 mV)) with a current resolution of 100 nA) and spectroradiometer (Konica–Minolta CS–2000 Tokyo, Japan) under ambient conditions. The spectroradiometer was also used to measure the EL spectrum and the xy chromaticity coordinates at a wavelength range of 380 nm to 780 nm, resolution of 0.9 nm/pixel, precision: ±0.3 nm/luminance range: 0.075 to 125,000 cd/m^2^ (measuring angle: 0.2°), accuracy: ±2%/chromaticity accuracy (over 1.25 cd/m^2^): x = ±0.0015, y = ±0.001). Particle images of QDs were obtained using a high-resolution transmission electron microscope (TEM, JEOL JEM–F200) (Tokyo, Japan) and the high-resolution cross-section images of the QD–LED device were collected using a Thermo Scientific Themis Z electron microscope (Waltham, MA, USA) operated at 80 kV and equipped with the X–ray Energy-Dispersive Energy Super–X detector used to analyze the elemental distribution. The cross–section specimens were prepared using a Thermo Scientific Helios UC instrument (Waltham, MA, USA). Atomic force microscopy (AFM) was done on a Nanosurf Easyscan–2 (cantilever ACLA–20, n–type silicon, spring constant 36–90 N/m, scan size of 5 × 5 µm) (Liestal, Switzerland). Thermogravimetric analysis (TGA) was carried out by a Mettler Toledo TGA 2 Thermogravimetric Analyzer (temperature range: 25 to 600 °C, mass resolution: 0.1 mg) (Columbus, AL, USA) at a heating rate of 10 K/min under N_2_ flow in aluminum crucibles.

## 3. Results and Discussion

### 3.1. Optical and Surface Properties of QD–OcSH, QD–PBSH and QD–TP

**Figure 3:**
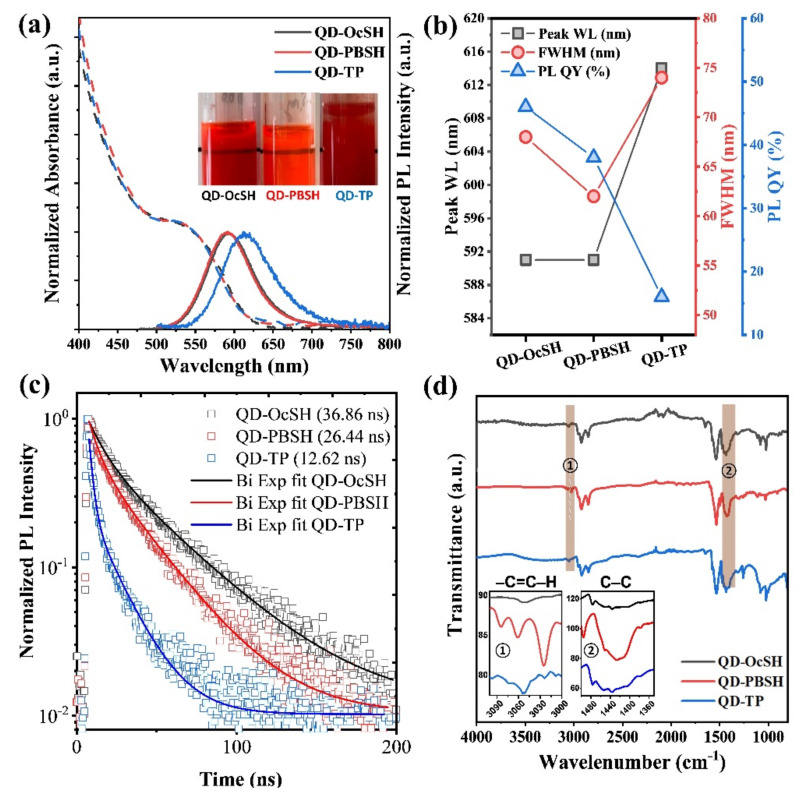
(**a**) Photoluminescence (PL) and ultraviolet–visible (UV–vis) absorption spectra including solubility in toluene (inset: a photograph of quantum dots (QDs) in toluene with 10 mg/mL concentration, which shows poor solubility of QD–TP), (**b**) optical properties, (**c**) decay curve of time resolved photoluminescence (TR–PL) at the emission peaks, and (**d**) Fourier transform infrared (FT–IR) spectrum of QD–OcSH (black), QD–PBSH (red), and QD–TP (blue).

Long–chain ligands in a range of 10–18 carbon atoms (C10–C18), such as oleic acid, are widely applied in nanomaterial synthesis because they lead to well–controlled size distribution, uniform dot-to-dot distance, and high optical performance [28,29]. However, these ligands have relatively low charge transport properties as compared to short ligand chains (<C8). Numerous studies have been performed to overcome this issue using QDs with short ligands in EL devices [30,31]. In particular, thiophenol (TP) derivates are known to have a negative dipole moment induced by their phenyl group (i.e., electron-donating group), which can facilitate the charge transport contrary to long-chain ligands such as oleic acid [32,33]. To investigate the influence of the ligand structure on the corresponding behavior in a QD–LED or QD/EBM nanohybrid material, we synthesized InP-based QDs with three different ligands: OcSH, PBSH and TP. Ligands were introduced via a simple in–situ ligand modification process in the last synthesis step as described in Section 2.5. All synthesized QDs had approximately the same size with 5.51 ± 0.64, 5.43 ± 1.00, and 5.4 ± 0.71 nm for QD–OcSH, QD–PBSH, and QD–TP, respectively (Figure S1). Basic photophysical characterization parameters before and after purification are shown in Table 2, Figure S2 and Figure 3a,b. Before purification, all QDs showed roughly the same emission wavelength of 591–593 nm as well as similar FWHM values of 67–69 nm. PL QY was determined to be 46% for QD–OcSH and 36% for QD–PBSH and QD–TP during synthesis, respectively.

However, despite the photophysical properties of the three QD samples being similar during synthesis, purification procedures had some influence on these characteristics as listed in Table 2. While the properties for QD–OcSH and QD–PBSH were stable during purification, QD–TP showed a significant redshift of the emission wavelength from 593 nm to 614 nm as well as a FWHM broadening from 69 nm to 74 nm and a significant drop in PL QY from 36% to 16% (see also Figure 3b).

The drop in PL QY in combination with the red–shifted EL spectrum and FWHM broadening during purification indicates that aggregation of QD–TP took place. The aggregation could also be observed in a toluene dispersion (inset Figure 3a). To compare the extent of ligand attachment, we conducted thermogravimetric analysis (TGA, Figure S3). The weight loss of the organic compounds for QD–OcSH, QD–PBSH, and QD–TP was determined to be 21.2%, 30.6%, and 13.3%, respectively. It was not possible to precisely identify the contribution of different ligands to the overall weight loss, but the TGA curves clearly confirm the contribution of different organic compounds, as indicated by two distinct weight drops. Overall, the TGA results support the observed behavior of the optical properties, showing that the QD–TP has the lowest level of ligand coverage. Two reasons could be responsible for this behavior. On the one hand, the bulkiness of TP ligands allows only fewer ligands to attach with their short distance between the benzene group and the QD surface, which makes the QD harder to stabilize in a dispersion. On the other hand, the phenyl groups are known to contribute to π–π stacking by chain–chain interdigitation in between molecules or particles with a crystalline-like properties [34,35], effectively linking different QDs and thus leading to aggregation. Based on this experience with the TP-ligands we designed PBSH consisting of an elongated n–alkyl chain between the thiol and the benzene group which provides a σ–bond rotation and bending freedoms, resulting in better surface coverage and thus solubility in the organic solvent. Furthermore, we investigated the time-resolved luminescence decay behavior of the three QD samples. Figure 3c shows the PL decay curves of QD–OcSH, QD–PBSH, and QD–TP measured at the peak emission wavelength. The decay curves were fitted with a bi-exponential function by following equation. $\;I\left(t\right)=\overset{n}{\underset{i=1}{\sum }}\alpha_{i}exp\left(-\frac{t}{\tau_{i}}\right)$, *n* = 2, where $I\left(t\right)\;$ is the transient light intensity and τ_i_ is the lifetime of each component. For all normalized decay curves, the fractional contribution of components are obtained by $f_{i}=\frac{\alpha_{i}\tau_{i}}{\sum_{j}\alpha_{j}\tau_{j}}\;\left(i=1,2\right)$. The intensity-weighted average lifetime was obtained by the following equation $\bar{\tau }=f_{1}\tau_{1}+f_{2}\tau_{2}$. The faster decay component τ_1_ is generally associated to the surface traps and the slower decay component τ_2_ to the excitonic transition in the InP core [36,37,38]. As listed in Table 3, the average lifetimes are decreasing in the order of QD–OcSH, QD–PBSH, and QD–TP with 32.95 ns, 25.02 ns, and 12.25 ns, respectively. QD–TP not only shows the shortest decay components with τ_1_ = 2.5 ns and τ_2_ = 18.1 ns but in addition τ_1_ also accounts for a large amount of the decay (37%). The fast decay behavior of QD–TP points to a strong contribution of surface defects to the relaxation pathways, further strengthening the argument of poor surface coverage of QD–TP.

To further investigate the extent to which each ligand is attached to the QD surface, FT–IR spectroscopy was undertaken. Distinctive peaks at 3000–3100 cm^−1^ and 1600–1450 cm^−1^ was observed, corresponding to aromatic –C=C–H stretching and C–C aromatic bending, respectively (Figure 3d) [39]. QD–PBSH showed the strongest aromatic absorption band followed by QD–TP and then QD–OcSH, which had, as expected, the lowest intensity due to incorporating no aromatic structure at all. These results further support the finding that an insufficient amount of TP ligands was attached to the QD surface which resulted in the poor solubility and quenching dominated the decay behavior of QD–TP.

### 3.2. The QD–LED Fabrications of QD–OcSH, QD–PBSH and QD–TP

**Figure 4:**
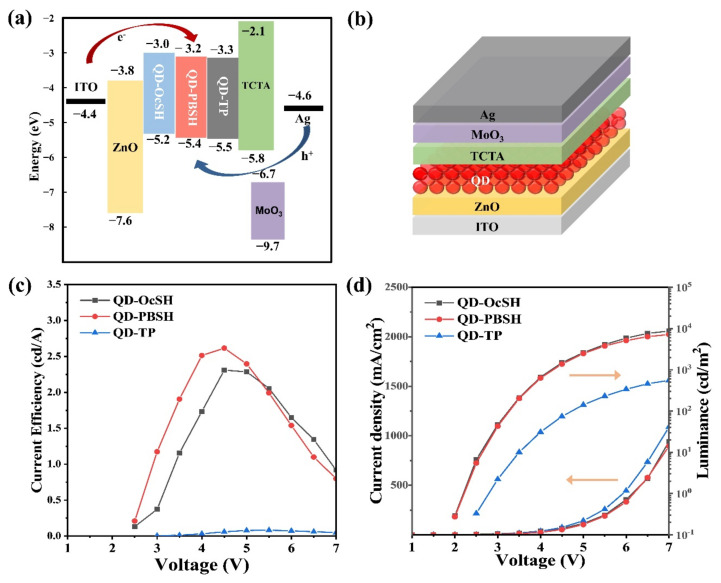
(**a**) A band diagram, (**b**) illustrated device structure, (**c**) current efficiency–voltage, and (**d**) J–V–L characteristics for the QD–LED fabricated with the active layer of QD–OcSH, QD–PBSH and QD–TP.

To determine the effect of modified ligands on the device performance, the three different QDs, QD–OcSH, QD–PBSH, and QD–TP, were applied in the inverted QD–LED structure as a single QD emission layer following the fabrication process described in Section 2.6. The band diagram and the device structure are shown in Figure 4a,b, respectively. The inverted structure (i.e., ITO/ZnO/QDs/TCTA/MoO_3_/Ag) was chosen to overcome one inherent drawback of the conventional device setup. The conventional structure uses organic hole transport materials, such as poly-TPD and PVK (e.g., ITO/PEDOT:PSS/poly–TPD or PVK/QDs/TPBi/Ca/Ag), which makes it difficult to establish an orthogonal solvent process between the HTL and the QD layer. Interface mixing between QDs and organic HTLs in a non-orthogonal solvent process during the spin-coating significantly influences the device performance in non-reproducible ways, which makes the interpretation of device performance data difficult [40]. In the inverted structure, ZnO NP were applied as ETL which enables an orthogonal solvent process [10,40,41]. 

The valence band maxima (VBM) of QD–OcSH, QD–PBSH, and QD–TP were investigated through photoelectron spectroscopy in air (PESA, AC–2) and found to be 5.2, 5.4, and 5.5 eV, respectively (Figure S4). Conduction band minima (CBM) were then calculated using the optical band gap values as determined by UV-Vis spectroscopy (3.0 eV, 3.2 eV, 3.3 eV, Figure 4a). These results are consistent with the tendency from previous reports where the negative dipole moment of the ligands was utilized to tune the energy level of QDs for better band alignment [24,42,43]. Moon et al. demonstrated that the energy level can be tuned by the thiol groups of TP, in which the negative dipole moment of the ligand was one of the influencing factors adjusting the level of VBM [15].

Nevertheless, the device with QD–TP showed extremely low current efficiency of 0.08 cd/A relative to other devices with QD–OcSH and QD–PBSH (i.e., 2.5 cd/A and 2.7 cd/A, respectively), as shown in Figure 4c. The maximum luminance of devices for QD–OcSH and QD–PBSH shows 16-fold and 13-fold higher values than QD–TP, as shown in the J–V–L curve of Figure 4d. In addition, the driving voltage measured at the luminance of 100 cd/m^2^ was increased to 4.6 V for QD–TP as compared to 3.2 V for both QD–OcSH and QD–PBSH. The turn-on voltage measured at 1 cd/m^2^ increased from 2.2 V for QD–OcSH and QD–PBSH to 2.8 V for QD–TP. In addition, the defect-related emission of the device with QD–TP in the range of 700–750 nm was significantly more pronounced compared to QD–OcSH and QD–PBSH (Figure S5a). This defect emission is in accordance with the results described in Section 3.1, where we concluded that the QD surface was not well passivated with TP ligands compared to QD–OcSH and QD–PBSH, which resulted in relatively low efficiency in the EL device for QD–TP. The slightly higher current efficiency from the device with QD–PBSH compared to QD–OcSH likely originates from the reduced CBM energy level. The electrons are more smoothly injected into the QD–PBSH active layer, due to the reduced electron injection barrier.

**Figure 5:**
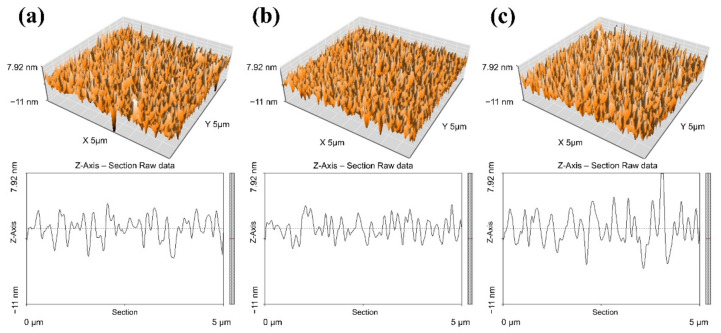
Atomic force microscopy (AFM) images of spin–coated QD film on poly-TPD film applied in the devices; (**a**) QD–OcSH (**b**) QD–PBSH (**c**) QD–TP.

To further evaluate how the different QD systems might influence the device performance, we investigated the surface morphology of QD films by AFM (Figure 5, Table 4). The root mean square surface roughness (R_q_) of films of QD–OcSH, QD–PBSH, and QD–TP were 2.15 nm, 1.84 nm, and 2.26 nm, respectively. Herein, QD–TP showed the highest R_q_ which can be attributed to the poor material solubility. In contrast, the R_q_ of QD–PBSH was relatively low, which means that the additive n–butyl chain between the thiol and benzene group led to a positive effect on the solubility of QDs and the surface morphology of the film. Nevertheless, the R_q_ value for QD–OcSH was surprisingly significantly closer to the value of QD–TP than to the value for QD–PBSH although OcSH provides a comparable flexibility to PBSH. Considering that the EL images of the QD–TP active layers are the only ones that show visible aggregations (Figure S5d), it is safe to assume that the R_q_ values are not reflecting the overall behavior of QD–TP during layer formation properly. Because the AFM pictures only show the morphology of a small part of the overall layer, it is very likely that the strongly localized aggregations are not necessarily reflected in the AFM pictures.

### 3.3. The QD–LED Fabrications of QD/Polymer Hybrids, QD–PBSH/PBCTA and QD–OcSH/PBCTA

**Figure 6:**
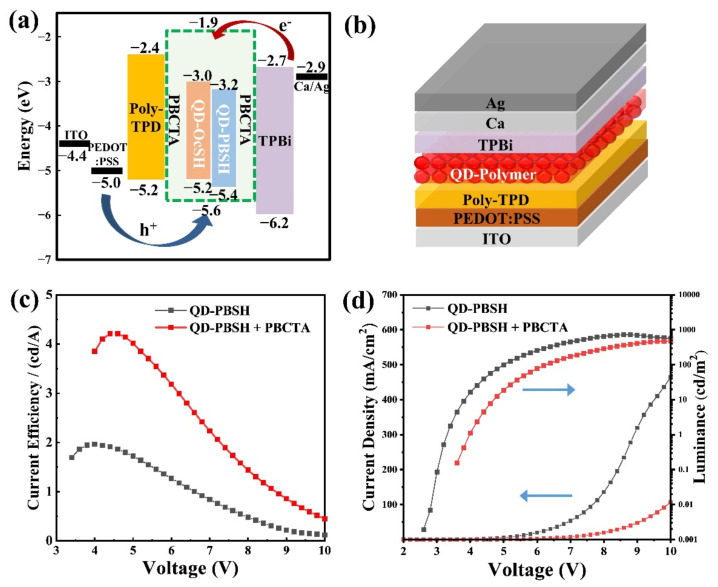
(**a**) Band diagram, (**b**) illustrated device structure, (**c**) current efficiency–voltage, and (**d**) J–V–L characteristics for the QD–LED fabricated with QD–PBSH/PBCTA hybrids as the active layer (the weight ratio of surface modified QDs to PBCTA is 1:1).

The QD–LED of the conventional structure was fabricated following Section 2.7, and we investigated the role of ligands on the QDs as well as possible synergistic effects with the side–chain polymer PBCTA as EBM regarding device performance. The band diagram, and device structure are shown in Figure 6a,b. As mentioned in the introduction, we aimed in particular at inducing an electron overcharge in the QD active layer by using TPBi as ETL in the conventional structure. The pre-synthesized PBCTA having a high LUMO level of −1.9 V (Table S1 and Figure S6) was employed as an EBM material for the QD/polymer hybrid active layer in the devices. In addition to that, the role of the surface ligands in the hybrid active layer was examined by applying QD–OcSH and QD–PBHS, respectively. QD–TP was not investigated because of the issues with dispersibility described in Section 3.1 and Section 3.2. The device performance for QD–PBSH with and without EBM are shown in Figure 6c,d. The device with the QD–PBSH/PBCTA nanohybrid material shows a twofold higher maximum current efficiency (4.2 cd/A @ 4.5 V) compared to the device without PBCTA (2 cd/A @ 4 V), as shown in Figure 6c. As shown in Figure S7a, a parasitic emission of poly-TPD at around 420 nm was observed in the device without PBCTA. The parasitic emission is induced by the overcharged QD emitting layer. The excess electrons in the QD layer are strongly delocalized and therefore easily transferred from the QD layer to the HTL, resulting in the recombination of electrons and holes in the poly-TPD layer [44,45]. One thing that is immediately noticeable for the devices with PBCTA is the improvement with regards to purity of the emission spectrum, as no or at least a strongly reduced parasitic emission at 420 nm was observed (Figure S7b). This behavior was accompanied by the suppression of current density in the entire driving voltage as shown in Figure 6d.

In contrast, the device with the QD–OcSH/PBCTA nanohybrid material showed no significant increase in maximum current efficiency (3.8 cd/A @ 4 V) compared to the device without PBCTA (4 cd/A @ 5.5 V), as shown at Figure S8b.

These results support two assertions. First, the electron blocking property of the EBM is confirmed to work as intended by the suppressed parasitic emission and current density. Second, the influence on device efficiency strongly indicates that there is indeed a synergistic effect between the PBSH ligands on the QD surface and the PBCTA, most probably through π–π interaction between the aromatic substructures of these molecules. On the contrary, there is no synergistic interaction between OcSH and PBTCA, due to OcSH providing no aromatic substructure.

**Figure 7:**
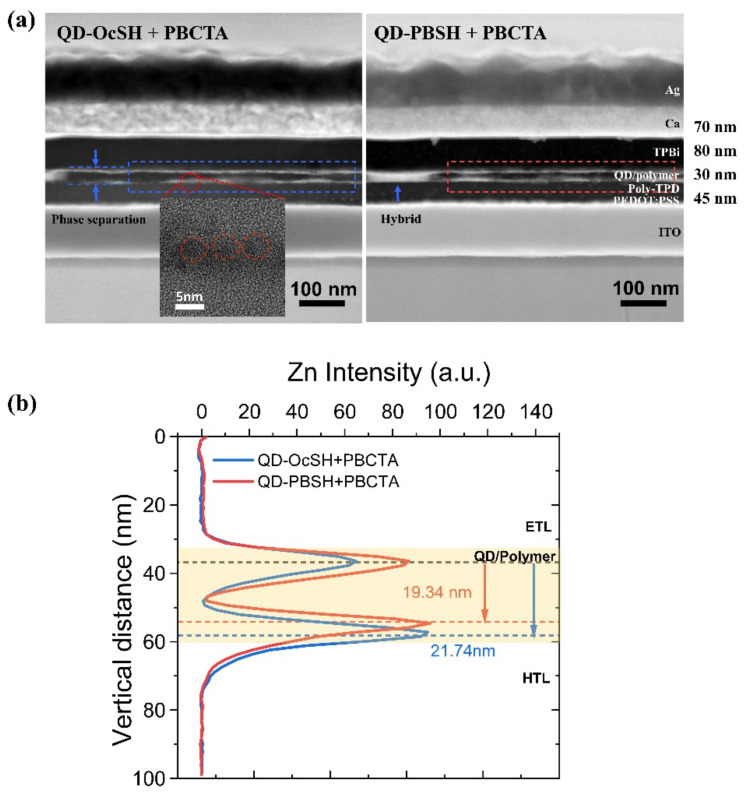
**(a)** The cross–sectional HAADF–STEM (high-angle annular dark field–scanning transmission electron microscopy) image of QD–LED with (left) QD–OcSH/PBCTA (the inset is a TEM image of QDs in the two distinct lines) and (right) QD–PBSH/PBCTA (**b**) the distribution of Zn atoms in the active layer as obtained from the Energy-Dispersive Energy Super–X (EDX) measurement.

To investigate the influence of the ligands on film morphology, we performed cross–sectional HAADF–STEM (high-angle annular dark field–scanning transmission electron microscopy). As shown in Figure 7a (left), two distinct lines were identified in the active layer, indicating a phase separation of QD–OcSH in the PBCTA matrix. The π–π interactions between QD–PBSH and PBCTA seem to reduce the phase separation as a larger hybrid area is observed in active layer with QD–PBSH (Figure 7a, right). The quantitative analysis of the degree of phase separation was conducted by scanning the elemental composition over the active layer. The distribution of Zn was determined in the marked 500 nm broad rectangular area, ranging vertically from the TPBi layer to the poly–TPD layer. Figure 7b shows the vertical Zn distribution in the scanned area. The peak distance indicates the degree of the phase separation for QD–OcSH/PBCTA and QD–PBSH/PBCTA, which were 21.34 nm and 19.34 nm, respectively. Accordingly, we can conclude that QD–PBSH reduced the phase separation by 11% relative to QD–OcSH, which was probably due to the π–π stacking effect. This morphological improvement of QD–PBSH/PBCTA correlates with the increased device performance, which was also reported previously by Fokina. A et al. [20]. Nevertheless, further studies are needed to characterize the influence of material interactions on the layer morphology and, consequently, on the device performance.

## 4. Summary and Conclusions

We have successfully demonstrated the application of concepts developed for Cd-based QDs and QD–LEDs in InP-based systems. In particular, we modified InP QDs with different thiol ligands incorporating an aromatic substructure. The TP ligand did not show the same behavior in the InP QD system as compared to the Cd-based QD system, due to low surface coverage of the ligand and poor solubility in organic solvents, resulting in poor QD–LED performance. We were able to address these issues by applying a different ligand design with the introduction of PBSH, which provides not only the thiol functionality but also an aromatic substructure with a saturated carbon chain in between, leading to more flexibility and reducing the space required for the ligands near the QD surface.

In addition, we were able to successfully prepare a new InP-QD/EBM nanohybrid material, utilizing PBCTA, which is known for its semiconducting and electron blocking properties. The electron-blocking properties as well as the synergistic effect between the aromatic substructures of PBCTA and PBSH enabled a better charge balance within the polymeric matrix and resulted in a twofold improved device efficiency. These results indicate the contribution of π–π interactions, inducing better charge transport within the polymeric matrix. These interactions between PBCTA and PBSH also lead to an improved morphology in the InP-QD/PBCTA nanohybrid active layer. Here, not only a decrease in phase separation was observed; additionally, we achieved a partial conservation of the InP-QD/PBCTA nanohybrid morphology even after device processing. These promising results point to the great potential of non-Cd QD/polymer nanohybrid composites as efficient emitting materials in inkjet-printed EL-QDLEDs. The nanohybrid material design contributes to a reduced number of printing steps and improved layer uniformity due to a potentially thicker active layer. In addition, various applications such as organic photovoltaics and light-emitting electrochemical cells will be interesting application areas for such nanohybrid material approaches.

## Figures and Tables

**Table 1:** The list and the abbreviations of chemicals.

Chemical	Abbreviation	Supplier	Purity
4–phenyl–1–butene	PhBe	Sigma Aldrich	99%
1–dodecanethiol	1–DDT	Sigma Aldrich	98%
1–octadecene,	1–ODE	Sigma Aldrich	90% technical grade
1-octanethiol	OcSH	Sigma Aldrich	≥98.5%,
1–octanoic acid	1–OctOAc	Sigma Aldrich	99%
2,2′,2″–(1,3,5–benzinetriyl)–tris(1–phenyl–1–H–benzimidazole,	TPBi	Lumtec	LT–E302
2,2′–azobis(2–methylpropionitrile)	AIBN	Sigma Aldrich	98%,
2–propanol	IPA	Carl Roth	99.5%
4,4′,4″–tris(carbazol–9–yl) triphenylamine	TCTA	Lumtec	
acetic acid glacial	AcOH	Sigma Aldrich	99%
acetone	AC	Carl Roth	99.9%
calcium	Ca	Balzers	
chloroform	CHCl_3_	Sigma Aldrich	≥99.8, anhydrous
ethanol	EtOH	Carl Roth	96%
ethyl acetate	EA	Acros organic	99.5%
indium (III) acetate	In(OAc)_3_	Sigma Aldrich	99.99% trace metals basis
magnesium sulfate	Mg_2_SO_4_	Sigma Aldirch	99.99% trace metals basis,
methanol	MeOH	Carl Roth	99%
molybdenum oxide	MoO_3_	Sigma Aldrich	99.99%
n–heptane	n–HEP	Sigma Aldrich	99%
poly–(N,N′–bis(4–butylphenyl)–N,N′–bis(phenyl)benzidine	PEDOT:PSS	Heraeus	CH8000
poly–(N,N′–bis(4–butylphenyl)–N,N′–bis(phenyl)benzidine)	poly-TPD	Solaris Chem Inc	SOL2420H
silver	Ag	Umicore	Lot # C000291487
tetrahyrofuran	THF	Sigma Aldrich	99.9%
thioacetic acid	TA	Alfa Aesar	96%
tributylphosphine	TBP	Sigma Aldrich	97%
trioctylphosphine	TOP	Sigma Aldrich	97%
tris(trimethylsilyl)phosphine	P(TMSi)_3_	Vezerf Laborsynthesen GmbH	97.5%
zinc acetate	Zn(OAc)_2_	Sigma Aldrich	99.99%,
zinc oxide zinc oxide	ZnO	Sigma Aldrich	99.99%

**Table 2:** Basic photophysical properties of synthesized InP/ZnSe/ZnS–QD samples before (b) and after (a) purification.

InP/ZnSe/ZnS–QD	Peak WL (nm) (b/a)	FWHM (nm) (b/a)	PL QY (%) (b/a)
QD–OcSH	591/591	68/68	46/46
QD–PBSH	591/592	67/62	36/38
QD–TP	593/614	69/74	36/16

**Table 3:** Summarized components of the bi-exponential fitting curve of decays for QD–OcSH, QD–PBSH and QD–TP.

QD	τ_1_ (ns)	α_1_	f_1_	τ_2_ (ns)	α_2_	f_2_	$\bar{\tau }$ (ns)
QD–OcSH	7.75	0.31	0.09	35.44	0.68	0.91	32.95
QD–PBSH	5.97	0.42	0.13	27.99	0.58	0.86	25.02
QD–TP	2.5	0.81	0.37	18.1	0.18	0.62	12.25

$\bar{\tau }\;$ = the intensity weighted average lifetime.

**Table 4:** Root mean square surface roughness (R_q_) data for QD film applied in the devices.

Film	QD–OcSH	QD–PBSH	QD–TP
R_q_ (nm)	2.15	1.84	2.26

## Data Availability

Not applicable.

## Supplementary Materials

The following are available online at https://www.mdpi.com/article/10.3390/nano12030408/s1, Figure S1: TEM image of QD–OcSH, QD-PBSH and QD-TP. The average diameter of QD–OCSH, QD–PBSH QD-TP is 5.41 ± 0.64, 5.43 ± 1.00, and 5.37 ± 0.71 nm (*n* = 200), respectively. Figure S2: Emission spectrum of (a) QD-OcSH, (b) QD-PBSH, and (c) QD-TP before and after purification. Figure S3: The thermogravimetric analysis (TGA) result of QD-OcSH, QD-PBSH and QD-TP. Figure S4: Photoelectron spectroscopy in Air (AC–2) measured throughout incident photon energy range of 3.4 eV to 6.2 eV. Figure S5: Normalized EL spectrum of QD–OcSH, QD–PBSH, and QD–TP with (a) linear and (b) logarithmic scale in the y–axis, and their EL images (scale bar: 0.5 mm) of (b) QD–OcSH, (c) QD–PBSH, and (d) QD–TP, respectively. Figure S6: Photoelectron spectroscopy in Air (AC–2) of PBCTA. Figure S7: EL emission of (a) QD–PBSH and (b) QD–PBSH + PBCTA in a logarithmic scale. Figure S8: (a) Current density luminance voltage (J-L-V) and (b) Current efficiency voltage graphs of the QD-LED device with QD–OcSH/PBCTA nanohybrid material; Table S1: HOMO level (measured by photoelectron spectroscopy), optical band gap and LUMO level (calculated from 1st excitation peak of absorption spectrum) of QDs and PBCTA.
