# Supplementary material for: Enhanced Electroluminescence via a Nanohybrid Material Consisting of Aromatic Ligand-Modified InP Quantum Dots and an Electron-Blocking Polymer as the Single Active Layer in Quantum Dot–LEDs
Source: Nanomaterials (Basel). 2022 Jan 26;12(3):408. doi: 10.3390/nano12030408 (PMC8838130; doi:10.3390/nano12030408)
Supplement: Supplementary file 1 [file nanomaterials-12-00408-s001.zip › nanomaterials-1569022-supplementary materials.pdf]

Supplementary Materials

Enhanced Electroluminescence via a Nanohybrid Material Consisting of Aromatic Ligand-Modified InP Quantum Dots and an Electron-Blocking Polymer as the Single Active Layer in Quantum Dot–LEDs

Hyung-Seok Choi [1], Silvia Janietz [1], Vladimir Roddatis [2], Andre Geßner [1], Armin Wedel [1], Jiyong Kim [1,*] and Yohan Kim [1,*]

[1] Functional Polymer Systems, Fraunhofer Institute for Applied Polymer Research (IAP), Geiselbergstrasse 69, 14476 Potsdam, Germany; hyung.seok.choi@iap.fraunhofer.de (H.-S.C.);
silvia.janietz@iap.fraunhofer.de (S.J.); andre.gessner@iap.fraunhofer.de (A.G.); armin.wedel@iap.fraunhofer.de (A.W.)

[2] GFZ German Research Center for Geosciences, Helmholtz Centre Potsdam, Telegrafenberg, 14473 Potsdam, Germany; vladimir.roddatis@gfz-potsdam.de

* Correspondence: jiyong.kim@iap.fraunhofer.de (J.K.); yohan.kim@iap.fraunhofer.de (Y.K.)

Table S1. HOMO level (measured by photoelectron spectroscopy), optical band gap and LUMO level (calculated from 1st excitation peak of absorption spectrum) of QDs and PBCTA.

QD	HOMO (eV)	LUMO (eV)	Optical bandgap (eV)
QD–OcSH	5.2	3.0	2.2
QD–PBSH	5.4	3.2	2.2
QD–TP	5.5	3.3	2.2
PBCTA	5.6	1.9	3.7

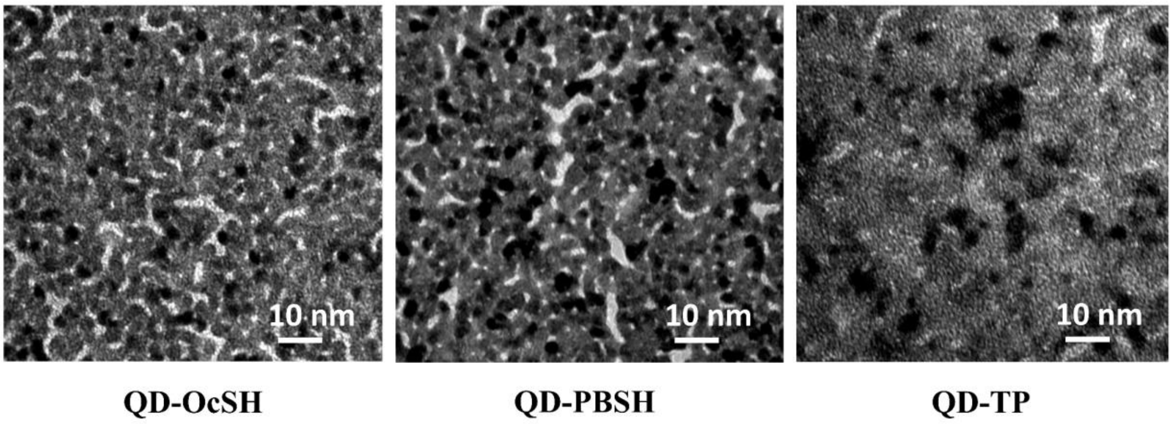

Figure S1. TEM image of QD–OcSH, QD–PBSH and QD–TP. The average diameter of QD–OCSH, QD–PBSH QD–TP is 5.41 ± 0.64, 5.43 ± 1.00, and 5.37 ± 0.71 nm (n = 200), respectively. Dot-to-dot distance of each QD was 1.27 nm and 1.61 nm and unmeasurable for the QD–TP aggregation.

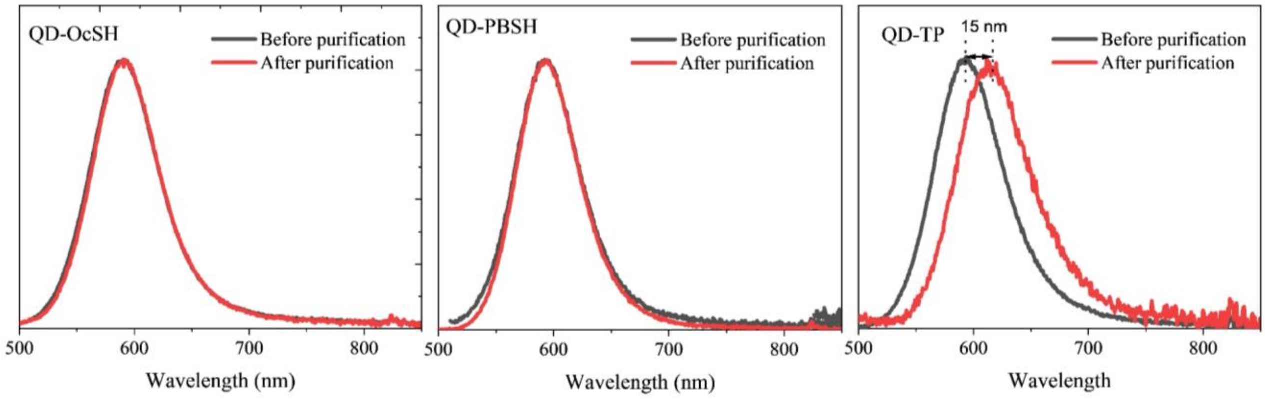

Figure S2. Emission spectrum of (**a**) QD–OcSH, (**b**) QD–PBSH, and (**c**) QD–TP before and after purification.

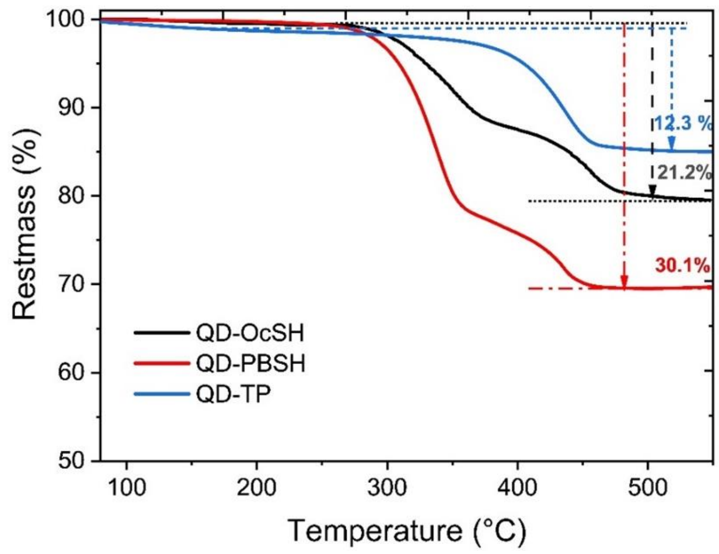

FigureS3. The thermogravimetric analysis (TGA) result of QD–OcSH, QD–PBSH and QD–TP.

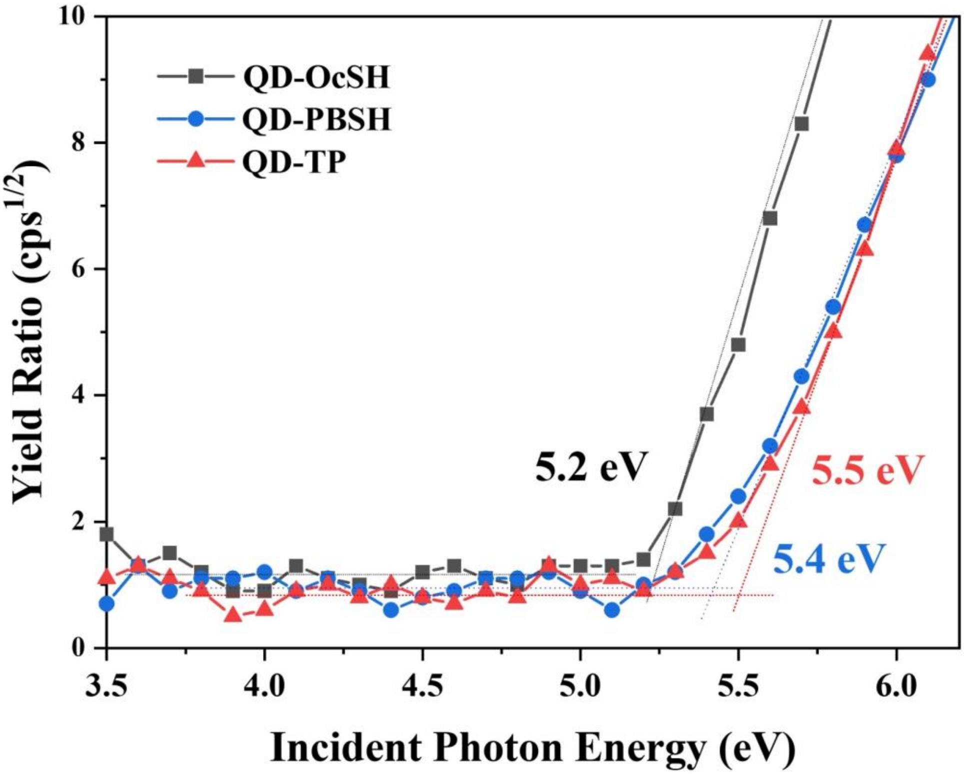

Figure S4. Photoelectron spectroscopy in Air (AC–2) measured throughout incident photon energy range of 3.4 eV to 6.2 eV.

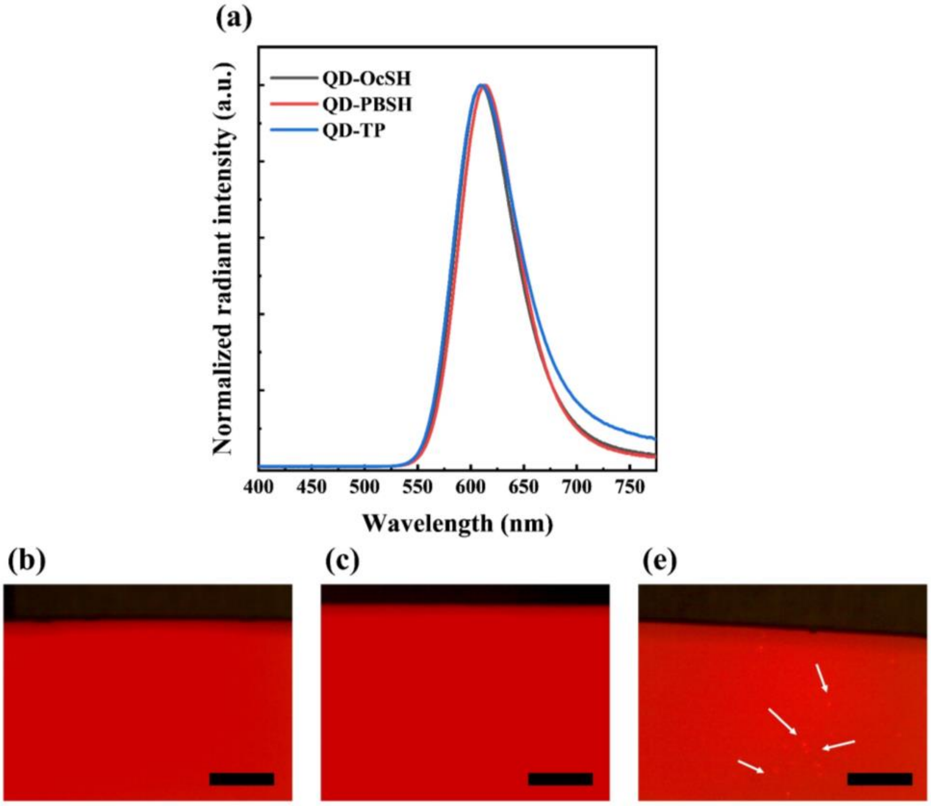

Figure S5. Normalized EL spectrum of QD–OcSH, QD–PBSH, and QD–TP with (**a**) linear and (**b**) logarithmic scale in the y–axis, and their EL images (scale bar: 0.5 mm) of (**b**) QD–OcSH, (**c**) QD–PBSH, and (**d**) QD–TP, respectively. No parasitic peaks at 500–540 nm originating from HTL (TCTA) were observed.

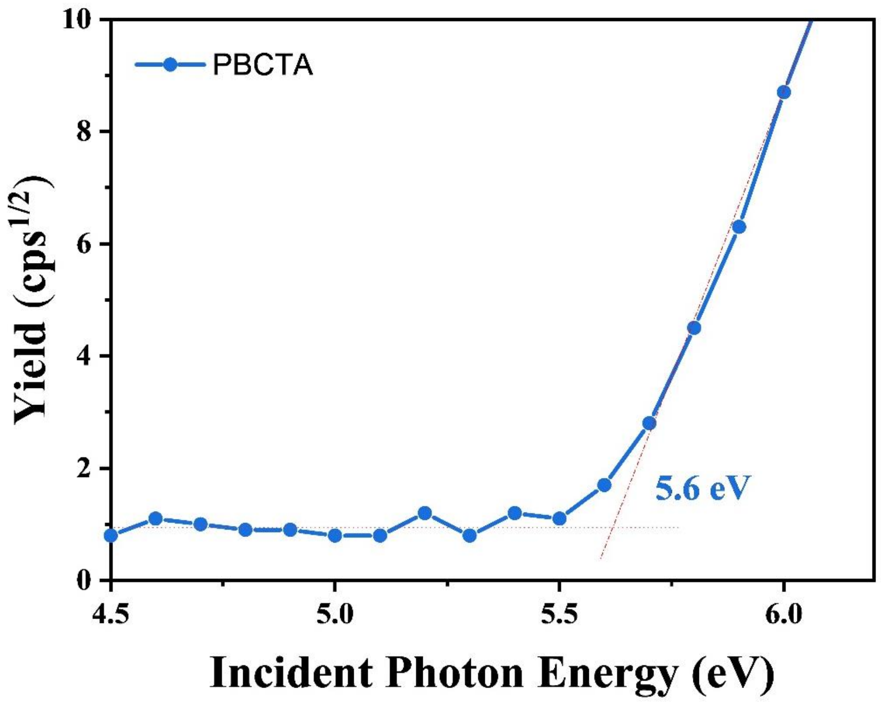

Figure S6. Photoelectron spectroscopy in Air (AC–2) of PBCTA.

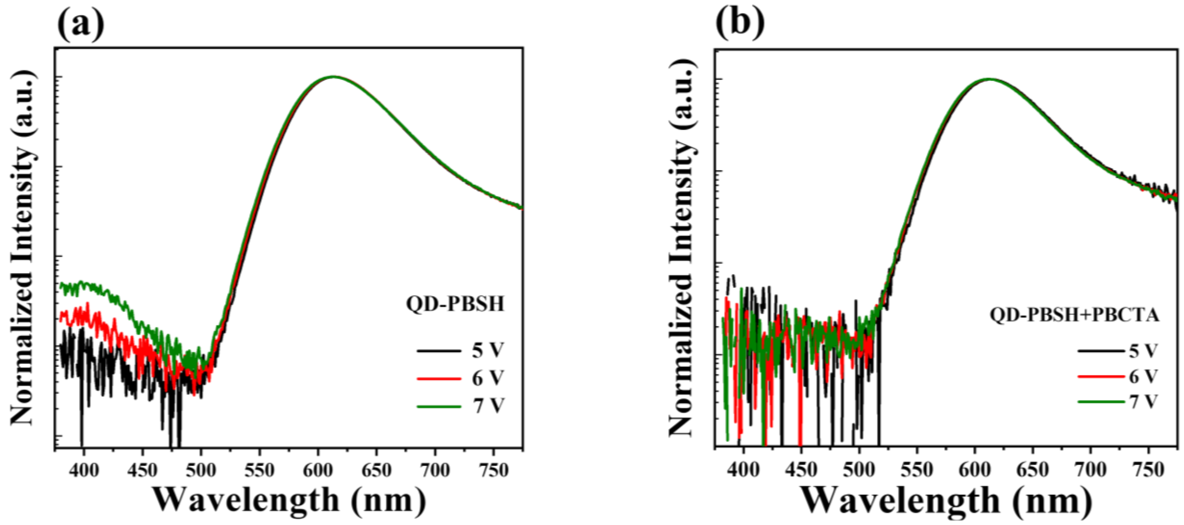

Figure S7. EL emission of (**a**) QD–PBSH and (**b**) QD–PBSH+PBCTA in a logarithmic scale. Parasitic emission of the HTL can only be observed at the peak around 420 nm for QD–PBSH.

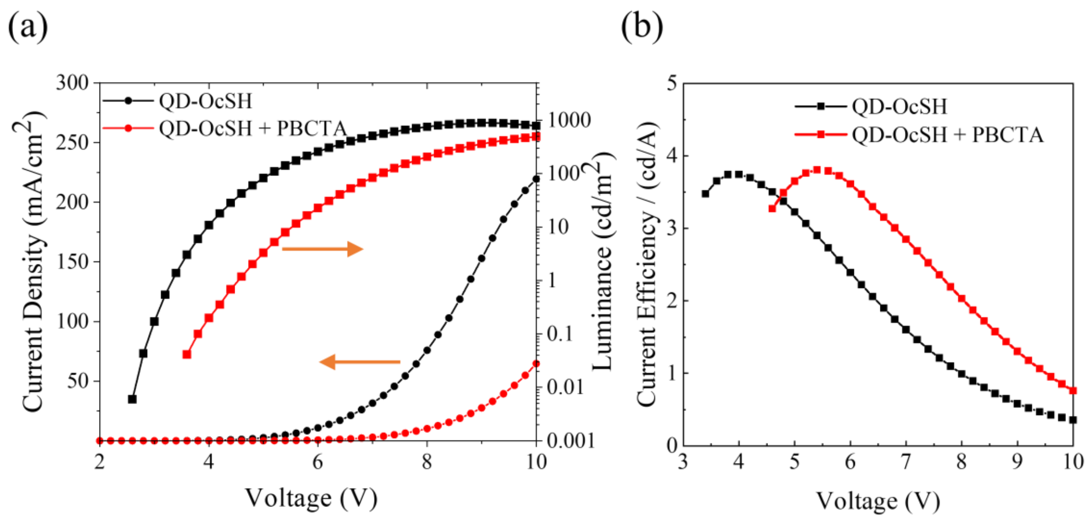

Figure S8. (**a**) Current density luminance voltage (J–L–V) and (**b**) Current efficiency voltage graphs of the QD–LED device with QD–OcSH/PBCTA nanohybrid material.
